# Interdisciplinary Rehabilitation for Concussion Recovery (i-RECOveR): protocol of an investigator-blinded, randomised, case series with multiple baseline design to evaluate the feasibility and preliminary efficacy of a 12-week treatment for persistent post-concussion symptoms

**DOI:** 10.1186/s40814-022-01153-6

**Published:** 2022-09-05

**Authors:** Jack V. K. Nguyen, Adam McKay, Jennie Ponsford, Katie Davies, Michael Makdissi, Sean P. A. Drummond, Jonathan Reyes, Catherine Willmott

**Affiliations:** 1 Turner Institute for Brain and Mental Health, School of Psychological Sciences, Monash University, GPO Box 1449, Melbourne, VIC 3001 Australia; 2 Monash-Epworth Rehabilitation Research Centre, Melbourne, Australia; 3 Neurological Rehabilitation Group, Melbourne, Australia; 4 Olympic Park Sports Medicine Centre, Melbourne, Australia; 5 Australian Football League, AFL House, 140 Harbour Esplanade, Docklands, Melbourne, VIC 3008 Australia

**Keywords:** Concussion, Mild traumatic brain injury, Interdisciplinary, Rehabilitation

## Abstract

**Background:**

Up to 25% of concussed individuals experience persistent post-concussion symptoms (PPCSs) which may interfere with the return to pre-injury activities and cause significant stress. Given that multiple etiological factors are thought to contribute to PPCSs, an interdisciplinary approach is recommended. This pilot study aims to primarily investigate the feasibility of a novel interdisciplinary treatment for PPCSs. Given this intervention is novel, uncertainty exists in terms of potential recruitment and retention rates, adverse events, and treatment adherence and fidelity. These factors will be explored to inform the feasibility of a phase-2 randomised controlled trial. Preliminary efficacy of this intervention will also be explored.

**Methods:**

Fifteen individuals with mild traumatic brain injury and PPCSs will receive up to 12 weeks of interdisciplinary treatments including psychology, physiotherapy, and medical interventions. Primary feasibility outcomes including data on recruitment and retention rates and treatment adherence will be explored descriptively. The cognitive therapy rating scale will be used to assess treatment fidelity. A single-case series with multiple baseline design will be used to explore preliminary efficacy. Participants will be randomly assigned to baseline phases of 2, 4, or 6 weeks. Regarding patient-centred secondary outcomes, the Rivermead Post-Concussion Symptoms Questionnaire will be assessed three times a week during baseline and treatment phases. Secondary outcomes also include measures of mood, sleep and fatigue, physical functioning, return to activity, and health-related quality of life. Patient-centred outcomes will be assessed at baseline, pretreatment, post-treatment, and one- and three-month follow-up. Thematic analysis of participant experiences will be explored through qualitative interviews.

**Discussion:**

Results from this trial will inform the feasibility and preliminary efficacy of this interdisciplinary concussion intervention and whether proceeding to a future definitive phase-2 randomised controlled trial is worthwhile. Understanding the end-user perspective of the treatment will also enable modifications to the treatment protocol for future trials to best suit the needs of individuals with PPCSs after mTBI. Outcomes from this trial can be directly translated into community rehabilitation programmes.

**Trial registration:**

ANZCTR, ACTRN12620001111965. Registered 27 October 2020, https://www.anzctr.org.au/Trial/Registration/TrialReview.aspx?id=379118

**Supplementary Information:**

The online version contains supplementary material available at 10.1186/s40814-022-01153-6.

## Introduction

Concussion/mild traumatic brain injury (mTBI) is the most prevalent form of traumatic brain injury (TBI), constituting 75–90% of all TBIs [27. , 53. , 59. ], with 1.6–3.8 million sport- and recreation-related concussions occurring annually in the USA alone [53. ]. There is limited literature on the incidence of concussion in Australia, although some have estimated that 170,000 concussions are sustained annually in Australia [108. ]. Concussion is defined by the World Health Organization and the American Congress of Rehabilitation Medicine as an acute brain injury that results from biomechanical forces (i.e. linear and rotational accelerations) to the head that causes functional disturbance within the brain. Common concussion symptoms include headaches, dizziness, nausea [28. , 43. , 58. , 83. ], sleep disturbance [48. ], fatigue [74. , 75. ], forgetfulness, poor concentration [6. ], irritability, anxiety, and depression [6. ].

Biomechanical forces of linear and rotational acceleration and deceleration have been identified as mechanisms underlying concussion [87. ]. These forces shift the brain within the skull and may result in a cascade of functional disturbances [31. ], inducing vestibular, oculomotor, autonomic, cognitive, and behavioural sequelae, collectively known as post-concussion symptoms [72. ]. Concussion sequelae are highly heterogeneous across individuals and may result in varying functional impairment and trajectories of recovery [20. ].

In adults, post-concussion symptoms generally resolve within 7–10 days [63. , 74. ]; however, up to 25% of concussed individuals experience delayed recovery which may interfere with the return to pre-injury activities (e.g. work, education, sport) and cause significant stress for months and sometimes years after injury [1. , 16. , 17. , 38. , 82. , 88. ]. Persistent post-concussion symptoms (PPCSs) have been described as the persistence of symptoms beyond the expected timeframe of recovery (i.e. > 4 weeks; [35. ]). PPCSs are thought to reflect a range of factors beyond the original injury to the brain including stress and anxiety [14. , 96. , 98. ] and physical factors such as concomitant injury to the neck and visual and/or balance systems [44. , 61. ]. Predictors of PPCS include premorbid factors such as previous loss of consciousness (LOC) and pre-injury psychological problems [76. ], with the latter often identified as the strongest predictor of outcome [80. , 107. , 111. ]. The presence of anxiety and depressive symptoms post-concussion is associated with the persistence of concussion symptoms more generally [39. , 74. , 75. , 114. ], as are coping styles, stress levels, and expectations of poor recovery [71. , 112. ]. Being a student, female sex, and older age and being injured in a motor vehicle accident have also been associated with poor recovery [23. , 77. , 98. ].

Research into treatments for PPCSs has primarily focussed on single discipline treatments targeting a single proposed mechanism (e.g. psychological, autonomic, vestibular). Psychoeducation interventions alone demonstrate limited efficacy in reducing concussion symptoms [106. ] and therefore may not be a panacea for concussion [97. ]. Given anxiety has been implicated in the maintenance of PPCSs, psychological interventions such as cognitive-behavioural therapy (CBT) represent a promising approach for the management of PPCSs [8. ]. Individualised CBT programmes for PPCSs have demonstrated efficacy in improving psychological distress [78. , 90. ] and quality of life [47. ] and reducing PPCSs [90. , 99. ]. Physiotherapy treatments including manual therapy (e.g. manipulation or stabilisation of the neck using hands rather than machines) [32. , 41. , 84. , 110. ], vestibular rehabilitation [9. , 36. , 37. , 94. ], oculomotor system interventions [19. , 91. , 92. ], and sub-symptom exercise therapy [30. , 54. , 55. ] have demonstrated efficacy in reducing impairment in their respective domains. In addition to psychological and physiotherapy management post-concussion, medical management may also be indicated [119. ], including prescribing pharmacology for the management of concussion sequelae such as post-traumatic headache [24. , 51. ].

Although there is evidence to support the efficacy of individual interventions in reducing PPCSs, accessing single-profession treatment is potentially limiting given that multiple etiological factors are often contributing to persisting post-concussion symptoms. It contrasts with clinical guidelines which emphasise a multidisciplinary approach to concussion management [26. , 60. , 61. ]. Specialised concussion services generally include a range of disciplines, primarily neuropsychology, physiotherapy, and medicine [3. , 4. , 119. ]. There is some evidence supporting the efficacy of multidisciplinary care models for PPCSs in military personnel [15. ] and adolescent [3. , 62. ] samples. There is also some evidence supporting interdisciplinary treatment of PPCSs in civilian adults [89. ]. Using a randomised clinical trial, Rytter et al. [89. ] demonstrated that a 22-week interdisciplinary programme with modules of psychoeducation and return to work delivered by a team comprised of neuropsychologists and physiotherapists and resulted in reductions in PPCSs immediately after treatment, with the maintenance of gains demonstrated at 6-month follow-up. They also found that reduction in PPCSs correlated with improved social functioning, increased activity and life satisfaction, and reduction in fatigue [89. ]. These findings demonstrate the potential utility of a collaborative and coordinated team-based approach to concussion management. Almost 20% of participants in this trial, however, were lost to follow-up and resulted in missing data for post-treatment and follow-up assessments [89. ]. This finding raises important practical concerns with regard to recruitment and the potential difficulty of retaining participants in a randomised treatment trial.

It is clear that further scientifically rigorous work in this area is warranted to evaluate the feasibility and efficacy of interdisciplinary interventions, evaluate dosage, and identify factors that contribute to the effectiveness of such interventions. There is also a need to control for the effects of spontaneous recovery when evaluating the potential efficacy of treatments for concussion. Research trials may address this by establishing baseline rates of symptoms over time before the introduction of treatments. This pilot study, using a single-case series design with multiple baselines, therefore aims to pilot and conduct a preliminary evaluation of an interdisciplinary intervention that incorporates expertise from neuropsychology, physiotherapy, and medicine to target the primary factors thought to contribute to PPCSs. Using a single-case series experimental design principally enables the examination of the appropriateness of the trial design (e.g. choice of outcome and measurement, recruitment protocol, adherence to treatment manual) to inform the design of a subsequent definitive randomised controlled trial as well as enabling the evaluation of preliminary efficacy of this novel treatment.

### Objectives

This study aims to investigate the feasibility and preliminary efficacy of interdisciplinary treatment for reducing post-concussion symptoms beyond 4-weeks post-injury. The primary objectives of the trial were as follows:

1. To evaluate the recruitment strategy and its effectiveness and whether eligibility criteria are too broad or strict
2. To quantify participant retention rates post-treatment and at 1- and 3-month follow-up timepoints
3. To examine therapist compliance to the manualised treatment protocol
4. To examine if the trial and/or treatments result in adverse effects

Secondary objectives are as follows:

1. To evaluate in a case series the preliminary efficacy of a pilot interdisciplinary intervention including neuropsychology, physiotherapy, and medical treatment in reducing post-concussion symptoms
2. To evaluate whether the treatment improves secondary patient-centred outcomes including goal attainment, mood, sleep and fatigue, physical functioning, return to activity, and health-related quality of life
3. To qualitatively explore the end-user perspective and acceptability of the treatment to facilitate modifications to the treatment protocol for future trials

## Methods

### Study design

The study will use a single-case series with nonconcurrent multiple baseline A-B design across subjects with 1- and 3-month follow-up post-treatment to examine treatment efficacy and maintenance of gains. The current multiple baseline design will have three tiers, with participants randomly assigned to one of three baselines (2, 4, or 6 weeks) to maximise experimental control [104. ]. The trial will be run as five three-tiered trials to enable replication of trials for the evaluation of external validity [104. ]. The risk of bias in N of 1 trials scale (ROBiNT) and the single-case reporting guideline in behavioural interventions (SCRIBE) will be used to ensure methodological rigour and reporting [102. , 103. ]. Individual and group level data from the single-case studies will be used to examine the feasibility of the intervention to inform the trial design of a phase 2 randomised controlled trial.

We will also explore participant experiences of the intervention using semi-structured interviews and analyse this data using thematic analysis.

### Study setting

This study will be conducted across three Melbourne-based community health centres with specialist services in concussion management.

### Participants

Participants will be 15 individuals aged between 16 and 70 with concussion classified as having a mild traumatic brain injury — Glasgow Coma Scale (GCS) score between 13 and 15, less than 30 min of loss of consciousness, less than 24 h of post-traumatic amnesia (PTA), and have persisting post-concussion symptoms (> 3 post-concussion symptoms rated 2 or above persisting for at least 1 month) [105. ] as indicated on the Rivermead Post-Concussion Symptoms Questionnaire (RPQ). Individuals must be at least 4 weeks post-injury but less than 12 months post-injury to ensure that symptoms are current and persistent [109. ]. Participants will also have sufficient proficiency in English to participate in the research assessments and treatment.

Exclusion criteria include moderate-severe TBI as indicated by *GCS* < 13, *PTA* > 24 h, positive findings on imaging, an acute psychiatric condition requiring intervention, significant neurological history, active substance abuse, significant medical history that would prevent completion of research assessment and/or treatments, and if they are having other concurrent treatment for concussion symptoms.

Sample size was determined based on current recommendations for single-case series methodology [49. , 104. ]. For multiple baseline designs to meet evidence standards, each phase (e.g. A — baseline or B — intervention) needs to have at least five data points. Furthermore, there needs to be at least six phases (three A-B tiers) in order to provide three replications of the experimental effect. Given these design standards, which suggest sample sizes in multiples of three, 15 participants will result in five replications of the three-tiered multiple baseline design, allowing for the evaluation of external validity [104. ].

### Recruitment

Participants endorsing PPCSs may be referred to the study by (a) the researchers through public advertisements or (b) clinicians through the three community health centres.

The potential participant will subsequently be screened by the research coordinator regarding the post-concussion symptoms they are experiencing and invited to participate in the research project if they satisfy selection criteria on the RPQ and do not meet the exclusion criteria. A record will be kept of the reason for declining to participate in the trial. Treatment as usual at the respective clinical services will be offered to those who decline to participate in the research trial if deemed appropriate. The nature of this treatment will be recorded.

Suitable participants will then meet with the research coordinator to complete written consent (see Additional file 1 for informed consent materials) and baseline measures. Demographic details (age, years of education, marital status, living situation, pre-injury, and current employment/study status), neurological history, drug and alcohol history, and current medications will be documented. Medical details including date and cause of injury, GCS scores, PTA duration, and routine clinical imaging findings as documented in medical files will also be obtained where available.

### Randomisation

A block randomisation method will be used to randomise participants into baseline duration phases of 2, 4, or 6 weeks. The following website will be used to facilitate randomisation https://www.random.org/lists/. Allocation concealment will be ensured by an independent research assistant to ensure that the research and treatment teams are adequately blinded to baseline phase duration. Allocation into baseline phases will be communicated to participants after all baseline (T1) measures have been completed.

### Outcome measures

#### Primary outcomes

Primary outcomes pertained to the feasibility of the trial and included measures of recruitment and retention rates, compliance to the manualised treatment protocol, and the presence of adverse events.

The number of participants referred to the trial and assessed for eligibility will be reported with reasons for exclusion documented. The total number of participants randomised will also be documented. Percentage of participants who completed post-treatment, 1-month follow-up, and 3-month follow-up assessments will also be reported with reasons for lost to follow-up also documented. Any adverse events occurring in the trial will be documented and described.

Compliance with the manualised treatment protocol will be assessed in terms of adherence and competency. Treatment adherence is operationalised as the extent of therapist adherence to the treatment protocol measured on a 7-point Likert scale where 1 represents “unacceptable” and 7 represents “excellent” adherence. Treatment competency will be assessed using the Cognitive Therapy Rating Scale (CTRS; [118. ]) and will reflect the extent to which the therapist implemented the interventions in a skilful manner. For physiotherapy and medical treatments, fidelity will be assessed by auditing clinical notes and evaluating whether the treatment modules have been completed.

#### Secondary patient-centred outcomes

Compliance with patient-centred outcomes will be assessed in terms of completion rates. The main patient-centred outcome measure will be post-concussion symptoms from the RPQ [45. ]. The RPQ assesses the severity of symptoms experienced after concussion with items categorised as physical symptoms (headaches, dizziness, nausea/vomiting, sleep disturbance, noise and light sensitivity, blurred vision, and double vision), cognitive symptoms (forgetfulness, poor concentration, slowed thinking), and behavioural symptoms (fatigue, irritability, tearfulness/depression, impatience, and restlessness). Comprised of 16, 5-point Likert-type items, participants will be asked to rate symptoms on a scale of 0 = “not experienced at all” to 4 = “a severe problem”. Higher scores on the RPQ indicate greater severity of post-concussion symptoms. As recommended by King et al. [45. ], RPQ items will be summed with ratings of 1 scored as 0 (as this response indicates that the symptom is experienced but is “no more of a problem than before the injury”), with total scores ranging from 0 to 64. There is evidence to suggest a unidimensional construct of symptoms, suggesting reliable calculation of total RPQ scores [5. ]. The RPQ demonstrates good test-retest and inter-rater reliability [45. ].

Other patient-centred outcomes of interest include measures of fatigue, sleep, health-related quality of life, depression, anxiety, and stress symptoms, vestibular, physiological, and cervical functioning, and postural stability. These outcomes will be assessed using questionnaires and physiotherapy outcome measures as summarised in Table 1.

**Table 1:** Secondary patient-centred outcome measures

***Questionnaires***	
**Brief Fatigue Inventory (BFI)** [64. ]	A 9-item fatigue scale to assess the severity and impact of fatigue on daily functioning in the past 24 h. The BFI has demonstrated reliable change after CBT for sleep disturbance and fatigue after TBI [67. ]
**Fatigue Severity Scale (FSS)** [50. ]	A 9-item general fatigue scale to assess behavioural consequences and impact of fatigue on daily functioning with demonstrated sensitivity to fatigue after TBI [120. ]
**Insomnia Severity Index (ISI)** [65. ]	A 7-item self-report questionnaire to assess the nature, severity, and impact of insomnia. The ISI has been used to evaluate CBT-I in adolescents with PPCSs, demonstrating clinically significant changes post treatment and at 4-week follow-up [109. ]
**SF-36 Health Survey (SF-36)** [115. ]	Measures the impact of the injury on the individual’s lifestyle from the perspective of the injured person. Widely used in TBI literature [73. ], the SF-36 has been validated in TBI samples and has shown good internal consistency [22. , 33. ]
**Depression Anxiety and Stress Scales-21 (DASS-21)** [57. ]	Measures symptoms of depression, anxiety, and stress experienced by the individual over the past week. The DASS-21 demonstrates very good internal consistency in TBI samples with the use of the Lovibond [57. ] 3-factor model validated in TBI rehabilitation [81. ]
**Brief Illness Perceptions Questionnaire (IPQ-B)** [13. ]	An eight-item measure of an individual’s cognitive perception of their illness, rated on an ordinal scale (0–10). Scores range from 0–80 with higher scores reflecting more negative perceptions of illness
***Physiotherapy outcome measures***	
**Vestibular/ocular motor screen (VOMS)** [66. ]	A screening tool developed to detect the signs and symptoms of concussion, testing for five areas of vestibular and ocular motor impairment including smooth pursuits, saccadic or rapid eye movements, near point of convergence, vestibular ocular reflex, and visual motor sensitivity. The VOMS demonstrates excellent internal consistency and sensitivity in identifying individuals with mTBI [66. ], with positive VOMS performances associated with delayed recovery [2. ]
**Balance Error Scoring System (BESS)** [34. ]	An objective measure of static postural stability. The BESS has demonstrated high reliability in samples of individuals with PPCSs [21. ]
**Buffalo Concussion Treadmill Test (BCTT)** [54. ]	Safe and validated test to monitor symptoms and physiological responses to graded exercise demands after mTBI [56. ]
**Flexion/rotation test of the cervical spine** [85. ]	Tests the integrity and range of the upper cervical spine. In this test, the cervical spine is fully flexed, to isolate movement to C1-C2, which has a unique ability to rotate in flexion. The procedure has high sensitivity and specificity to detect the presence or absence of cervical joint dysfunction in neck pain and headache patients [95. ]
**Smooth pursuit neck torsion test (SPNTT)** [85. ]	A test for identifying cervical pathology through dizziness and altered ocular movements with the neck in a position of torsion vs a midline position. Sensitive in whiplash injuries
***Neuropsychological assessment measures***	
**Test of premorbid functioning (TOPF;** [117. ])	Measures premorbid intellectual ability
**Oral Symbol Digit Modalities Test (Oral SDMT**; [100. ])	Assesses information processing speed. The oral version was chosen over the pencil-and-paper version to suit telehealth administration if required during the COVID-19 pandemic
**WAIS-IV Digit Span Forward** [116. ]	Assesses basic verbal attentional capacity
**WAIS-IV Digit Span Backward **[116. ]	Assesses working memory
**Oral Trail Making Test Ricker and Axelrod** [86. ]	Assesses information processing speed and divided attention. The oral version was chosen over the pencil-and-paper version to suit telehealth administration if required during the COVID-19 pandemic
**Rey Auditory Verbal Learning Test (RAVLT**; [93. ])	A measure of verbal learning and memory

As part of the intervention, participants will work with the clinician to develop treatment goals. These goals will be assessed and monitored using goal attainment scaling (GAS; [46. ]). GAS goals provide an opportunity for person-centred and participant-generated outcome and is a method of qualitatively evaluating the extent to which a participant’s personalised goals of return to activity are attained during the course of the intervention. Setting GAS goals with individuals with acquired brain injury has been demonstrated to be feasible [10. ]. GAS is a valid measure for use in rehabilitation with good sensitivity to change [40. ]. Goal attainment is scored on a 5-point scale with 0 representing that the goal was achieved as expected. Positive scores indicate that participants achieved more than expected with +1 indicating the goal was achieved “somewhat more” than expected and +2 indicating “much more” than expected. Negative scores indicate that participants achieved less than expected, with −1 and −2 representing “somewhat less” and “much less”, respectively.

#### Additional baseline cognitive measures

To characterise baseline cognitive functioning of the sample to inform their treatment, participants will complete a brief neuropsychological assessment with a research assistant (summarised in Table 1).

### Assessment timepoints

**Fig. 1:**
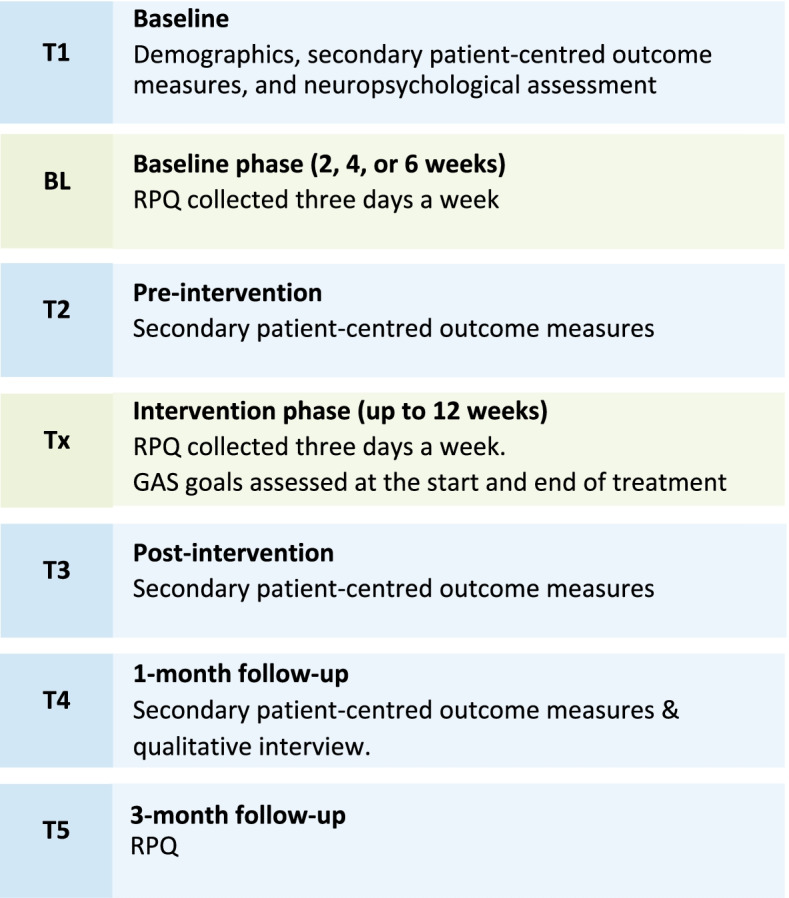
Schedule of study assessments

Post-allocation, participants will be seen across five timepoints where they will be asked to complete secondary patient-centred outcomes. See below summary of the assessment timepoints and Table 2 for participant schedule adapted from the Standard Protocol Items: Recommendations for Interventional Trials (SPIRIT) recommendations [18. ]. We have also included a visual representation of this as Fig. 1. See Additional file 2 for SPIRIT checklist. To improve participant retention at post-treatment follow-up timepoints, participants will be reminded of the follow-up appointments by their treating clinicians toward the end of the treatment phase. To promote engagement at 1-month follow-up, participants will be reimbursed with a AU $50 gift card for completing an interview at 1-month follow-up. To limit attrition, the 3-month follow-up was designed to be less onerous for participants.

**Table 2:** Schedule of enrolment, interventions, and assessments

	Study period								
	Enrolment	Allocation	Post-allocation						
Timepoint^**a**^	***-t***_***1***_		***t***_***1***_	***BL***	***t***_***2***_	***Tx***	***t***_***3***_	***t***_***4***_	***t***_***5***_
**Enrolment**									
**Eligibility screen and demographics interview**	X								
**Informed consent**	X								
**Allocation**		X							
**Interventions**									
***i-RECOveR***						X			
**Assessments**									
***RPQ***			X	X	X	X	X	X	X
***BFI***			X		X		X	X	
***FSS***			X		X		X	X	
***ISI***			X		X		X	X	
***SF-36***			X		X		X	X	
***DASS-21***			X		X		X	X	
***IPQ-B***			X		X		X	X	
***WAIS-IV DSF, DSB***			X						
***RAVLT***			X						
***ORAL SDMT***			X						
***ORAL TMT—A and B***			X						
***TOPF***			X						
***VOMS***			X		X		X	X	
***SPNTT***			X		X		X	X	
***Flexion rotation***			X		X		X	X	
***BESS***			X		X		X	X	
***BCTT***			X		X		X	X	
***Qualitative interview***								X	

^a^*−t*_1_ enrolment, *t*_1_ baseline assessment, *BL* baseline phase (of either 2, 4, or 6 weeks), *t*_2_ end of baseline assessment, *T*x treatment phase, *t*_3_ post-treatment assessment, *t*_4_ 1-month post-treatment follow-up, *t*_5_ 3-month post-treatment follow-up

#### T1: Start of baseline (1-day before 2, 4, or 6 weeks of baseline phase)

At baseline assessment, participants will complete the RPQ and secondary outcome questionnaires online using REDCap with a research assistant present to answer any queries the participant may have. They will also complete the neuropsychological assessment measures and physiotherapy outcome measures on this occasion.

##### Baseline phase

Once randomised and informed of length of baseline phase, participants will complete the RPQ three times a week on a Monday, Wednesday, and Friday. They will receive the RPQ as a REDCap link via email at 8:00 am and will receive a reminder email at 5:30 pm on the same day if they have not completed the RPQ. If participants do not complete the RPQ after the email reminder, they will be sent a follow-up text message reminder at 8:00 pm. If no response is made after the 8:00 pm reminder, this data point will be noted as incomplete.

#### T2: End of baseline (within 1 week of completing the baseline phase)

Participants will receive a REDCap link and will be asked to complete the questionnaires at the end of their baseline phase. They will also complete physiotherapy outcome measures at this time in an appointment at the clinic with research physiotherapists (i.e. those therapists collecting outcome measures are not the same as those providing treatment).

##### Intervention phase

As in the baseline phase, participants will complete the RPQ three times a week on Monday, Wednesday, and Friday and will be provided with reminders as outlined above.

#### T3: Post-intervention (within 1-week post-intervention)

As in the T2 assessment, participants will receive a REDCap link to complete questionnaires. They will also see a research physiotherapist at this time to complete secondary outcome measures.

#### T4: 1-month post-treatment follow-up

As in the T2 and T3 assessments, participants will complete questionnaires online and physiotherapy outcome measures with a research physiotherapist. To elicit participant experiences of their persistent post-concussion symptoms and of the interdisciplinary intervention, semi-structured interviews will be conducted.

#### T5: 3-month post-treatment follow-up

Participants will be sent a REDCap link and asked to complete the RPQ.

### Assessment and treatment standardisation

Baseline neuropsychological assessment will be completed by a trained neuropsychology PhD student using standardised administration and instructions. At all timepoints, questionnaires will be completed online using a REDCap survey. Physiotherapy measures will be completed in person with a trained physiotherapist using established protocols for each outcome measure to ensure standardised assessment.

Psychology treatments will be delivered by registered psychologists with skills in CBT and knowledge of concussion and will complete regular supervision from authors experienced in the treatment (AM, CW, JP). Adherence to the treatment manual and competency in CBT will be assessed and reported. Physiotherapy treatments will be administered by physiotherapists who work in the neurological and vestibular population and are trained in concussion management. They will be overseen with regular supervision by an author (KD) with experience in concussion management. Medical treatments will be delivered by a team of medical practitioners working with established clinic protocols and evidence-based treatments for concussion.

### Blinding

To ensure that the research and treatment teams are adequately blinded to baseline phase duration of each participant, an independent research assistant will coordinate all research assessment and treatment assessment appointments beyond the initial baseline assessment. The research coordinator will complete recruitment and screening interviews with potential participants and then organise neuropsychology and physiotherapy baseline assessments, communicating these dates to an independent research assistant. This research assistant will then inform participants of their baseline phase duration at the completion of all baseline assessments. They will also coordinate end of baseline phase assessments and treatment appointments without disclosing baseline phase duration to treating clinicians. Baseline neuropsychological assessments will be conducted by the research coordinator and not the treating neuropsychologist. Likewise, there will be separate physiotherapists for research assessments (research physiotherapists) and for the provision of treatment (treating physiotherapist). At the end of the trial, given the nature of the single-case series data analysis, the trial coordinator and lead author (JN) will be unblinded to conduct statistical analyses.

### Interdisciplinary treatment programme

The i-RECOveR treatment manual was developed by the investigators in 2020 through consultations with collaborators from the three Melbourne-based community health centres. This treatment programme includes individualised and coordinated psychology, physiotherapy, and medical interventions.

**Fig. 2:**
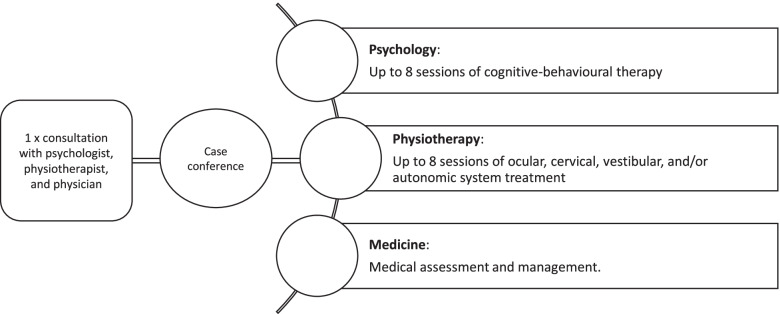
Concurrent interdisciplinary intervention

All participants will have an initial consultation with a neuropsychologist, physiotherapist, and sports medicine physician. In these consultations, the clinicians will obtain a history from the participant and, through interview, establish the participant’s presenting complaints. Following these consultations, the clinicians will meet via case conference to triage the participant’s treatment needs, establish GAS goals, and develop a treatment plan for the participant. As part of our interdisciplinary intervention, participants with PPCSs will be offered eight sessions each of psychological and physiotherapy treatment and medical management over 12 weeks as required based on the initial consultations (Fig. 2).

The minimum intervention required to qualify as having received interdisciplinary treatment is attendance at all initial consultations with the three treating clinicians. Ongoing management (up to eight sessions of physiotherapy, up to eight sessions of psychology, medical management as needed) will differ across participants depending on clinical needs and will be documented. All treatments offered and received will be documented.

#### Psychological intervention

As part of the intervention, participants will attend an initial consultation with a psychologist. The psychologist will refine case understanding and formulation, provide psychoeducation on concussion, and discuss treatment structure. The psychological intervention will be oriented toward a cognitive-behavioural framework as described by Beck [7. ] and will be adapted from previous manuals developed by Ferguson and Mittenberg [29. ] and Silverberg et al. [99. ] and comprises the following components: goal setting, psychoeducation regarding concussion and recovery, activity scheduling, addressing beliefs and expectations, anxiety management training, and sleep intervention.

#### Physiotherapy intervention

Participants will also attend an initial consultation with a physiotherapist where they will receive a full impairment-based assessment of the visual, vestibular, and cervical system based on their history and subjective presentation. Based on this initial assessment, the physiotherapist will develop an individualised treatment plan for the patient depending on cervical, vestibular, ocular, and physiological outcome measures (described below). Participants will also receive education on the physical presentation of concussion and an educational handout. Up to eight sessions of physiotherapy treatment, which includes movement and exercise, manual therapy, education, and advice regarding physiological changes after concussion, will be offered. Treatment will target the visual, balance, neck, and autonomic systems and primarily consist of exercises which improve and resolve symptoms. The physiotherapist will track progress over time to evaluate participant response to treatment and adjust the treatment plan if required.

#### Medical intervention

In addition to psychology and physiotherapy interventions, medical management will be provided to participants. Participants will attend an initial consultation with a physician. The physician will collect a detailed history of the concussion, any past history (including number, recency, and duration of symptoms), and the presence of any concussion modifiers (i.e. predictors of prolonged symptoms such as learning difficulties, history of migraine, mood disorder) [42. ]. This assessment will also involve assessment of post-concussion symptoms and neurological examination. Medical treatment will be directed at the domains that are most commonly affected by concussion. Medications including simple analgesics, amitriptyline, anti-depressants, anti-migraine medication, and cervical spine injections may be prescribed depending on the clinical presentation [69. ]. Medical management of PPCSs will comprise ongoing consultation with the physician as required, which may include pharmacological management of symptoms as well as advice regarding return to activities (e.g. work, sport, school).

#### Adapting to telehealth during the COVID-19 pandemic

Participants will be recruited during the COVID-19 pandemic. The aim is for all treatment and research assessments to be completed face to face, but given the expected interruptions due to the state of Victoria COVID-19 lockdowns, the following adaptions are designed to facilitate ongoing data collection during the pandemic. Baseline neuropsychological assessments (T1) and 1-month follow-up post-treatment interviews (T4) will be completed via videoconference (e.g. Zoom) during the lockdowns which restricts travel for non-essential reasons. Under the state of Victoria COVID-19 lockdown restrictions, individuals may leave their house to seek treatment, therefore permitting travel to complete i-RECOveR intervention sessions. Neuropsychology intervention sessions may be completed via telehealth using the videoconferencing platform Zoom. Physiotherapy and medical services will remain face to face as permitted. For all face-to-face appointments, treatments will be conducted in accordance with established COVIDSafe plans for each clinic.

#### Treatment cessation

As part of the interdisciplinary treatment programme, clinicians will have ongoing communication with each other throughout the participant’s individualised treatment (communication between clinicians will be documented). Participants may be discharged from treatment early based on clinical opinion. They may be discharged from one discipline before the other (e.g. if physical issues resolve, they would be discharged from physiotherapy yet may continue psychological intervention to address persisting cognitive, mood, or behavioural issues).

#### Relevant concomitant care and interventions

Participants may undergo concomitant care and interventions for comorbid conditions. Concomitant treatments will be documented at the start of the trial. Parallel treatments for concussion will not be permitted during the trial.

### Qualitative guide to explore participant experiences of i-RECOveR

This mixed-methods study will also qualitatively explore participant experiences of i-RECOveR.

#### Participant experience

To elicit the participant’s experience of their PPCSs and the intervention, the following interview guide will be utilised in semi-structured interviews:

1. What was your *experience* of the intervention?
2. What were the *most helpful elements* of the intervention?
3. What were the *least helpful elements* of the intervention?
4. How has the intervention changed the way you think about your concussion?
5. Compared to how you were before the intervention, what *effects have you noticed on your life* since completing the intervention?
6. What effects have you noticed on your *primary complaint* (e.g. mood/cognition/headaches)?
7. Was there anything else you would like to say about the intervention?

### Data analysis plan

#### Data management plan

All data will be deidentified and stored securely on REDCap. Prior to the commencement of data collection, a REDCap database will be established. Where applicable, range checks for data values will be embedded into the database. To mitigate missing data, all items of a measure must have a value entered for the measure to be marked complete. Throughout the trial, data audits will be completed by two researchers, and all items for questionnaires will be double scored.

##### AIM 1: Feasibility of i-RECOveR

Data on recruitment and retention rates, protocol compliance, adverse events, treatments received, and treatment acceptability will be collected and analysed descriptively to inform guidelines for progressing the pilot trial to a larger phase 2 trial [25. ]. Adequate completion rates (i.e., 75%), limited adverse events, and signs of preliminary efficacy will inform whether the trial will progress to a randomised controlled design.

**Treatment fidelity**

Psychology treatment sessions will be audio recorded to allow for analysis of treatment fidelity. Recordings of 10% of the sessions will be rated by an independent psychologist, evaluating adherence to the treatment protocol. For physiotherapy and medical treatments, fidelity will be assessed by auditing clinical notes and evaluating whether the treatment modules have been completed.

##### AIM 2: Preliminary efficacy of i-RECOveR

**Post-concussion symptom severity**

Participant-reported post-concussion symptoms (RPQ) will be graphed to allow for visual inspection using GraphPad Prism 8. Systematic visual analysis will be conducted in line with established guidelines proposed by Spriggs, Lane, and Gast [101. ] and Lane and Gast [52. ], incorporating both within- and between-phase analyses to evaluate trend, level, and stability of data. Trend will be assessed using the split-middle method using median values [52. ].

Statistical analysis of single-case experimental data (i.e. total RPQ scores collected three times a week) will be conducted to augment visual analysis to provide more robust conclusions about the dataset. Planned comparisons between baseline and intervention phases will be conducted using the non-overlap method, Tau-U [68. ]. Tau-U is a nonparametric approach, appropriate for small data sets, which controls for improving trends (whether linear, curvilinear, or mixed) in the baseline phase and autocorrelation to generate an effect size [113. ]. An online Tau-U calculator (http://www.singlecaseresearch.org/calculators/tau-u) will be used to check for trend in the baseline, adjust for it where necessary, and calculate contrasts between phases [113. ]. This calculator will also be used to test omnibus statistics.

**Secondary patient-centred outcomes**

Regardless of whether participants receive physiotherapy, psychological, or medical treatments beyond the three initial consultations, they will complete all secondary outcome measures according to an intention to treat analysis. GAS goals will be descriptively explored with any changes considered clinically significant [70. ]. Given this is a pilot investigation of the preliminary efficacy of this 12-week intervention, the focus for the secondary outcome measures will be on clinically significant change. Secondary outcome measures (BFI, FSS, ISI, DASS-21, IPQ-B, VOMS, BESS, SPNTT, flexion rotation, BCTT) will also be explored descriptively with 95% confidence intervals to explore clinically significant changes across the four assessment timepoints.

##### AIM 3: Qualitative analysis of participant experience of i-RECOveR

All participants will complete an interview using a semi-structured interview guide with the aim of producing an analysis of participants’ experience of the interdisciplinary intervention, exploring what elements they found helpful and those they found unhelpful, as well as exploring the impact(s) of the intervention on their symptoms and daily functioning. All interviews will be audio recorded. Recordings will be transcribed by author JN using NVivo software (version 12; [79. ]). An orthographic/verbatim notation system will be used as outlined by Braun and Clarke [12. ].

An inductive approach to analysis of the interview transcripts will be completed with codes derived from the data as per the six-phase thematic analysis approach outlined by Braun and Clarke [11. ]. The initial phase will require familiarisation of the data which will be undertaken during the interview and transcription stages. Following this, documentation of initial patterns and codes will be completed. The data will then be systematically recoded. Codes will then be categorised to produce themes and subthemes. Resultant themes will then be reviewed to ensure that they reflect the coded texts and are representative of the broader data set. Themes will then be defined and named before the manuscript is drafted. Regular investigator meetings, cross-checking of transcripts, codes, and revisions of themes will be planned to ensure rigour of the analysis.

### Ethics and dissemination

The trial has been registered with the Australian New Zealand Clinical Trials Registry (Trial ID: ACTRN12620001111965). Ethics approval has been obtained from the Monash University Human Research Ethics Committee (MUHREC number: 23005). Written parental consent and participant assent will be obtained for all participants aged 16 to 17. Written consent will be obtained for all participants aged 18 and older. Participants can withdraw from the study at any time. CBT, physiotherapy, and medical consultations have been demonstrated to be safe and tolerable for individuals with PPCSs. Although there are no known risks of participating in these interventions, it is possible that participants may find some of the content during the intervention difficult and/or emotionally challenging. The physiotherapy interventions may induce symptoms (e.g. dizziness), however, this will be done in a controlled environment under the care of trained physiotherapists. Research and clinical staff involved in the project are experienced in assessing and managing potential adverse events associated with this treatment and associated outcome measures should they arise.

Results from this study will be disseminated in peer-reviewed journals and will be submitted to local and international conferences. Given the study design, results could potentially be identifiable to those who know the cases well (e.g. doctors, family members). Identifying information will be limited as much as possible.

## Discussion

Persisting concussion symptoms after mTBI can disrupt return to education, employment, leisure activities including sport, social participation, and family relationships and can be a source of significant distress [1. , 16. , 17. , 38. , 82. , 88. ], requiring interdisciplinary support. Despite this, there are few studies worldwide evaluating interdisciplinary concussion interventions in adults, and evidence regarding the efficacy of these interventions is sparse. There is good indication that this may in fact be the best practice, but more evidence is required. In undertaking this research, we aim to first evaluate the treatment in a series of case studies and qualitatively evaluate participant experiences of the i-RECOveR treatment programme.

The results from this trial will inform the study design of a larger definitive phase-2 randomised controlled trial in the following ways:

1. A fully powered randomised controlled trial of treatment for PPCSs has not been evaluated in an Australian context. Given this, there remains uncertainty regarding potential recruitment and retention rates. This trial will provide an opportunity to pilot the proposed recruitment strategy and make amendments if necessary to facilitate participant recruitment and engagement.
2. Given that i-RECOveR is a new treatment, assessment of compliance (i.e. adherence and competency) to the treatment protocol is critical before progressing to a larger trial. By closely examining treatment recordings and compliance ratings, investigators will be able to identify areas of the manual that need further attention with regard to therapist training and supervision.
3. Patient-centred outcomes will also be explored as part of this pilot investigation. It is hoped that this trial will serve to also inform the appropriateness of these measures for future trials as well as providing pilot data to inform the potential compliance rates associated with each measure. Given the study will use a robust single-case experimental design, signs of preliminary efficacy can be examined to explore whether proceeding to a phase-2 RCT is worthwhile.
4. By undertaking qualitative interviews with participants in the pilot study, the end-user perspective of treatment will be elicited. It is anticipated that the responses will inform the unique experiences of i-RECOveR for each participant to identify aspects of the trial and treatment that worked or did not work. By understanding the end-user perspective, the pilot study will enable the adaptation of the treatment manual to better address the needs of individuals with PPCSs after mTBI.

The main trial will aim to assess the effectiveness of the intervention as well as providing insights into the participants’ experience of an interdisciplinary concussion intervention. Outcomes from this trial will be directly translated into the community. This research will evaluate treatments that are currently provided by the clinicians, with a focus on interdisciplinary management, and it is hoped that this project will eventually establish an evidence-based treatment programme for concussion.

### Trial status

Recruitment commenced in January 2021, and it is still ongoing.

## Supplementary Information

**Additional file 1.** Informed consent materials.

**Additional file 2.** SPIRIT Checklist.

## Data Availability

Not applicable.

## References

[1] Al Sayegh A, Sandford D, Carson AJ (2010). Psychological approaches to treatment of postconcussion syndrome: a systematic review. J Neurol Neurosurg Psychiatry.

[2] Anzalone AJ, Blueitt D, Case T, McGuffin T, Pollard K, Garrison JC (2017). A positive vestibular/ocular motor screening (VOMS) is associated with increased recovery time after sports-related concussion in youth and adolescent athletes. Am J Sports Med.

[3] Bailey C, Meyer J, Briskin S, Tangen C, Hoffer SA, Dundr J (2019). Multidisciplinary concussion management: a model for outpatient concussion management in the acute and post-acute settings. J Head Trauma Rehabil.

[4] Baker JG, Willer BS, Leddy JJ (2019). Integrating neuropsychology services in a multidisciplinary concussion clinic. J Head Trauma Rehabil.

[5] Balalla S, Krägeloh C, Medvedev O, Siegert R (2020). Is the Rivermead Post-Concussion Symptoms Questionnaire a aeliable and valid measure to assess long-term symptoms in traumatic brain injury and orthopedic injury patients? A novel investigation using rasch analysis. Neurotrauma Rep.

[6] Barker-Collo S, Jones K, Theadom A, Starkey N, Dowell A, McPherson K (2015). Neuropsychological outcome and its correlates in the first year after adult mild traumatic brain injury: a population-based New Zealand study. Brain Inj.

[7] Beck AT (1979). Cognitive therapy and the emotional disorders.

[8] Bergersen K, Halvorsen JØ, Tryti EA, Taylor SI, Olsen A (2017). A systematic literature review of psychotherapeutic treatment of prolonged symptoms after mild traumatic brain injury. Brain Inj.

[9] Bhattacharyya N, Gubbels SP, Schwartz SR, Edlow JA, El-Kashlan H, Fife T (2017). Clinical practice guideline: benign paroxysmal positional vertigo (update). Otolaryngol Head Neck Surg.

[10] Bouwens SF, Van Heugten CM, Verhey FR (2009). The practical use of goal attainment scaling for people with acquired brain injury who receive cognitive rehabilitation. Clin Rehabil.

[11] Braun V, Clarke V (2006). Using thematic analysis in psychology. Qual Res Psychol.

[12] Braun V, Clarke V (2013). Successful qualitative research: a practical guide for beginners.

[13] Broadbent E, Petrie KJ, Main J, Weinman J (2006). The brief illness perception questionnaire. J Psychosom Res.

[14] Bryant RA, O’donnell ML, Creamer M, McFarlane AC, Clark CR, Silove D (2010). The psychiatric sequelae of traumatic injury. Am J Psychiatr.

[15] Caplan B, Bogner J, Brenner L, Janak JC, Cooper DB, Bowles AO (2017). Completion of multidisciplinary treatment for persistent postconcussive symptoms is associated with reduced symptom burden. J Head Trauma Rehabil.

[16] Carroll L, Cassidy JD, Peloso P, Borg J, Von Holst H, Holm L (2004). Prognosis for mild traumatic brain injury: results of the WHO collaborating centre task force on mild traumatic brain injury. J Rehabil Med.

[17] Cassidy JD, Cancelliere C, Carroll LJ, Côté P, Hincapié CA, Holm LW (2014). Systematic review of self-reported prognosis in adults after mild traumatic brain injury: results of the International Collaboration on Mild Traumatic Brain Injury Prognosis. Arch Phys Med Rehabil.

[18] Chan AW, Tetzlaff JM, Gøtzsche PC, Altman DG, Mann H, Berlin JA, Moher D (2013). SPIRIT 2013 explanation and elaboration: guidance for protocols of clinical trials. Br Med J.

[19] Ciuffreda KJ, Rutner D, Kapoor N, Suchoff IB, Craig S, Han M (2008). Vision therapy for oculomotor dysfunctions in acquired brain injury: a retrospective analysis. Optometry.

[20] Collins MW, Kontos AP, Okonkwo DO, Almquist J, Bailes J, Barisa M (2016). Statements of agreement from the targeted evaluation and active management (TEAM) approaches to treating concussion meeting held in Pittsburgh, October 15-16, 2015. Neurosurgery.

[21] Cushman D, Hendrick J, Teramoto M, Fogg B, Bradley S, Hansen C (2018). Reliability of the balance error scoring system in a population with protracted recovery from mild traumatic brain injury. Brain Inj.

[22] Diaz AP, Schwarzbold ML, Thais ME, Hohl A, Bertotti MM, Schmoeller R (2012). Psychiatric disorders and health-related quality of life after severe traumatic brain injury: a prospective study. J Neurotrauma.

[23] Dischinger PC, Ryb GE, Kufera JA, Auman KM (2009). Early predictors of postconcussive syndrome in a population of trauma patients with mild traumatic brain injury. J Trauma Acute Care Surg.

[24] Dubrovsky AS, Friedman D, Kocilowicz H (2014). Pediatric post-traumatic headaches and peripheral nerve blocks of the scalp: a case series and patient satisfaction survey. Headache.

[25] Eldridge SM, Chan CL, Campbell MJ, Bond CM, Hopewell S, Thabane L, Lancaster GA. PAFS consensus group. CONSORT 2010 statement: extension to randomised pilot and feasibility trials. BMJ. 2016;355:i5239. 10.1136/bmj.i5239. 10.1136/bmj.i5239 PMC5076380 27777223

[26] Ellis MJ, Leddy J, Willer B (2016). Multi-disciplinary management of athletes with post-concussion syndrome: an evolving pathophysiological approach. Front Neurol.

[27] Faul M, Coronado V. Epidemiology of traumatic brain injury. In: Handbook of clinical neurology, vol. 127. The Netherlands: Elsevier; 2015. p. 3–13. 10.1016/B978-0-444-52892-6.00001-5 25702206

[28] Faux S, Sheedy J (2008). A prospective controlled study in the prevalence of posttraumatic headache following mild traumatic brain injury. Pain Med.

[29] Ferguson RJ, Mittenberg W. Cognitive—behavioral treatment of postconcussion syndrome. In: Sourcebook of psychological treatment manuals for adult disorders. United States: Springer; 1996. p. 615–55.

[30] Gagnon I, Galli C, Friedman D, Grilli L, Iverson GL (2009). Active rehabilitation for children who are slow to recover following sport-related concussion. Brain Inj.

[31] Giza CC, Hovda DA (2014). The new neurometabolic cascade of concussion. Neurosurgery.

[32] Gross AR, Hoving JL, Haines TA, Goldsmith CH, Kay T, Aker P (2004). A Cochrane review of manipulation and mobilization for mechanical neck disorders. Spine.

[33] Guilfoyle MR, Seeley HM, Corteen E, Harkin C, Richards H, Menon DK, Hutchinson PJ (2010). Assessing quality of life after traumatic brain injury: examination of the short form 36 health survey. J Neurotrauma.

[34] Guskiewicz KM (2003). Assessment of postural stability following sport-related concussion. Curr Sports Med Rep.

[35] Henry LC, Elbin RJ, Collins MW, Marchetti G, Kontos AP. Examining recovery trajectories after sport-related concussion with a multimodal clinical assessment approach. Neurosurg. 2016;78(2):232–41. 10.1227/NEU.0000000000001041 PMC4833014 26445375

[36] Hillier S, Hollohan V (2007). Vestibular rehabilitation for unilateral peripheral vestibular dysfunction (Cochrane review).

[37] Hilton MP, Pinder DK. The Epley (canalith repositioning) manoeuvre for benign paroxysmal positional vertigo. Cochrane Database Syst Rev. 2004;(12). 10.1002/14651858.CD003162.pub3 PMC11214163 25485940

[38] Hiploylee C, Dufort PA, Davis HS, Wennberg RA, Tartaglia MC, Mikulis D (2017). Longitudinal study of postconcussion syndrome: not everyone recovers. J Neurotrauma.

[39] Hou R, Moss-Morris R, Peveler R, Mogg K, Bradley BP, Belli A (2012). When a minor head injury results in enduring symptoms: a prospective investigation of risk factors for postconcussional syndrome after mild traumatic brain injury. J Neurol Neurosurg Psychiatry.

[40] Hurn J, Kneebone I, Cropley M (2006). Goal setting as an outcome measure: a systematic review. Clin Rehabil.

[41] Hurwitz EL, Carragee EJ, van der Velde G, Carroll LJ, Nordin M, Guzman J (2009). Treatment of neck pain: noninvasive interventions: results of the Bone and Joint Decade 2000–2010 Task Force on Neck Pain and Its Associated Disorders. J Manip Physiol Ther.

[42] Iverson GL, Gardner AJ, Terry DP, Ponsford JL, Sills AK, Broshek DK, Solomon GS (2017). Predictors of clinical recovery from concussion: a systematic review. Br J Sports Med.

[43] Junn C, Bell KR, Shenouda C, Hoffman JM (2015). Symptoms of concussion and comorbid disorders. Curr Pain Headache Rep.

[44] Kennedy E, Quinn D, Chapple C, Tumilty S (2019). Can the neck contribute to persistent symptoms post concussion? A prospective descriptive case series. J Orthop Sports Phys Ther.

[45] King N, Crawford S, Wenden F, Moss N, Wade D (1995). The Rivermead Post Concussion Symptoms Questionnaire: a measure of symptoms commonly experienced after head injury and its reliability. J Neurol.

[46] Kiresuk TJ, Sherman RE (1968). Goal attainment scaling: a general method for evaluating comprehensive community mental health programs. Community Ment Health J.

[47] Kjeldgaard D, Forchhammer HB, Teasdale TW, Jensen RH (2014). Cognitive behavioural treatment for the chronic post-traumatic headache patient: a randomized controlled trial. J Headache Pain.

[48] Kontos AP, Sufrinko A, Sandel N, Emami K, Collins MW (2019). Sport-related concussion clinical profiles: clinical characteristics, targeted treatments, and preliminary evidence. Curr Sports Med Rep.

[49] Kratochwill TR, Hitchcock JH, Horner RH, Levin JR, Odom SL, Rindskopf DM, Shadish WR (2013). Single-case intervention research design standards. Remedial Spec Educ.

[50] Krupp LB, LaRocca NG, Muir-Nash J, Steinberg AD (1989). The fatigue severity scale: application to patients with multiple sclerosis and systemic lupus erythematosus. Arch Neurol.

[51] Kuczynski A, Crawford S, Bodell L, Dewey D, Barlow KM (2013). Characteristics of post-traumatic headaches in children following mild traumatic brain injury and their response to treatment: a prospective cohort. Dev Med Child Neurol.

[52] Lane JD, Gast DL (2014). Visual analysis in single case experimental design studies: brief review and guidelines. Neuropsychol Rehabil.

[53] Langlois JA, Rutland-Brown W, Wald MM (2006). The epidemiology and impact of traumatic brain injury: a brief overview. J Head Trauma Rehabil.

[54] Leddy JJ, Willer B (2013). Use of graded exercise testing in concussion and return-to-activity management. Curr Sports Med Rep.

[55] Leddy JJ, Kozlowski K, Donnelly JP, Pendergast DR, Epstein LH, Willer B (2010). A preliminary study of subsymptom threshold exercise training for refractory post-concussion syndrome. Clin J Sport Med.

[56] Leddy J, Hinds AL, Miecznikowski J, Darling S, Matuszak J, Baker JG (2018). Safety and prognostic utility of provocative exercise testing in acutely concussed adolescents: a randomized trial. Clin J Sport Med.

[57] Lovibond SH, Lovibond PF (1996). Manual for the depression anxiety stress scales.

[58] Lucas S, Hoffman JM, Bell KR, Dikmen S (2014). A prospective study of prevalence and characterization of headache following mild traumatic brain injury. Cephalalgia.

[59] Maas AI, Menon DK, Adelson PD, Andelic N, Bell MJ, Belli A (2017). Traumatic brain injury: integrated approaches to improve prevention, clinical care, and research. Lancet Neurol.

[60] Makdissi M, Schneider KJ, Feddermann-Demont N, Guskiewicz KM, Hinds S, Leddy JJ (2017). Approach to investigation and treatment of persistent symptoms following sport-related concussion: a systematic review. Br J Sports Med.

[61] Marshall CM, Vernon H, Leddy JJ, Baldwin BA (2015). The role of the cervical spine in post-concussion syndrome. Phys Sportsmed.

[62] McCarty CA, Zatzick D, Stein E, Wang J, Hilt R, Rivara FP (2016). Collaborative care for adolescents with persistent postconcussive symptoms: a randomized trial. Pediatrics.

[63] McCrory P, Meeuwisse W, Dvorak J, Aubry M, Bailes J, Broglio S, Vos PE. Consensus statement on concussion in sport—the 5th international conference on concussion in sport held in Berlin, October 2016. Br J Sports Med. 2017;51(11):838–47. 10.1136/bjsports-2017-097699 28446457

[64] Mendoza TR, Wang XS, Cleeland CS, Morrissey M, Johnson BA, Wendt JK, Huber SL (1999). The rapid assessment of fatigue severity in cancer patients: use of the brief fatigue inventory. Cancer.

[65] Morin CM, Belleville G, Bélanger L, Ivers H (2011). The Insomnia Severity Index: psychometric indicators to detect insomnia cases and evaluate treatment response. Sleep.

[66] Mucha A, Collins MW, Elbin R, Furman JM, Troutman-Enseki C, DeWolf RM (2014). A brief vestibular/ocular motor screening (VOMS) assessment to evaluate concussions: preliminary findings. Am J Sports Med.

[67] Nguyen S, McKay A, Wong D, Rajaratnam SM, Spitz G, Williams G (2017). Cognitive behavior therapy to treat sleep disturbance and fatigue after traumatic brain injury: a pilot randomized controlled trial. Arch Phys Med Rehabil.

[68] Parker RI, Vannest KJ, Davis JL, Sauber SB (2011). Combining nonoverlap and trend for single-case research: Tau-U. Behav Ther.

[69] Patricios J, Fuller GW, Ellenbogen R, Herring S, Kutcher JS, Loosemore M (2017). What are the critical elements of sideline screening that can be used to establish the diagnosis of concussion? A systematic review. Br J Sports Med.

[70] Perdices M (2005). How do you know whether your patient is getting better (or worse)?: a user’s guide. Brain Impair.

[71] Polich G, Iaccarino MA, Kaptchuk TJ, Morales-Quezada L, Zafonte R (2020). Nocebo effects in concussion: is all that is told beneficial?. Am J Phys Med Rehabil.

[72] Polinder S, Cnossen MC, Real RG, Covic A, Gorbunova A, Voormolen DC (2018). A multidimensional approach to post-concussion symptoms in mild traumatic brain injury. Front Neurol.

[73] Polinder S, Haagsma JA, van Klaveren D, Steyerberg EW, Van Beeck EF (2015). Health-related quality of life after TBI: a systematic review of study design, instruments, measurement properties, and outcome. Popul Health Metrics.

[74] Ponsford JL, Ziino C, Parcell DL, Shekleton JA, Roper M, Redman JR (2012). Fatigue and sleep disturbance following traumatic brain injury—their nature, causes, and potential treatments. J Head Trauma Rehabil.

[75] Ponsford J, Cameron P, Fitzgerald M, Grant M, Mikocka-Walus A, Schönberger M (2012). Predictors of postconcussive symptoms 3 months after mild traumatic brain injury. Neuropsychology.

[76] Ponsford J, Nguyen S, Downing M, Bosch M, McKenzie JE, Turner S (2019). Factors associated with persistent post-concussion symptoms following mild traumatic brain injury in adults. J Rehabil Med.

[77] Ponsford J, Willmott C, Rothwell A, Cameron P, Kelly A-M, Nelms R (2000). Factors influencing outcome following mild traumatic brain injury in adults. J Int Neuropsychol Soc.

[78] Potter SD, Brown RG, Fleminger S (2016). Randomised, waiting list controlled trial of cognitive–behavioural therapy for persistent postconcussional symptoms after predominantly mild–moderate traumatic brain injury. J Neurol Neurosurg Psychiatry.

[79] QSR International Pty Ltd. NVivo (Version 12). 2018. https://www.qsrinternational.com/nvivo-qualitative-data-analysis-software/home.

[80] Rabinowitz AR, Li X, McCauley SR, Wilde EA, Barnes A, Hanten G (2015). Prevalence and predictors of poor recovery from mild traumatic brain injury. J Neurotrauma.

[81] Randall D, Thomas M, Whiting D, McGrath A (2017). Depression anxiety stress scales (DASS-21): factor structure in traumatic brain injury rehabilitation. J Head Trauma Rehabil.

[82] Rees RJ, Bellon ML (2007). Post concussion syndrome ebb and flow: longitudinal effects and management. NeuroRehabilitation.

[83] Register-Mihalik JK, Mihalik JP, Guskiewicz KM (2008). Balance deficits after sports-related concussion in individuals reporting posttraumatic headache. Neurosurgery.

[84] Reid SA, Rivett DA, Katekar MG, Callister R (2008). Sustained natural apophyseal glides (SNAGs) are an effective treatment for cervicogenic dizziness. Man Ther.

[85] Reneker JC, Moughiman MC, Cook CE (2015). The diagnostic utility of clinical tests for differentiating between cervicogenic and other causes of dizziness after a sports-related concussion: an international Delphi study. J Sci Med Sport.

[86] Ricker JH, Axelrod BN (1994). Analysis of an oral paradigm for the Trail Making Test. Assessment.

[87] Rowson S, Duma SM, Beckwith JG (2012). Rotational head kinematics in football impacts: an injury risk function for concussion. Ann Biomed Eng.

[88] Ruff RM (2011). Mild traumatic brain injury and neural recovery: rethinking the debate. NeuroRehabilitation.

[89] Rytter HM, Westenbaek K, Henriksen H, Christiansen P, Humle F (2019). Specialized interdisciplinary rehabilitation reduces persistent post-concussive symptoms: a randomized clinical trial. Brain Inj.

[90] Scheenen ME, Visser-Keizer AC, de Koning ME, van der Horn HJ, van de Sande P, van Kessel M (2017). Cognitive behavioral intervention compared to telephone counseling early after mild traumatic brain injury: a randomized trial. J Neurotrauma.

[91] Scheiman M, Cotter S, Kulp MT, Mitchell GL, Cooper J, Gallaway M (2011). Treatment of accommodative dysfunction in children: results from an random clinical trial. Optom Vis Sci.

[92] Scheiman M, Gwiazda J, Li T. Non-surgical interventions for convergence insufficiency. Cochrane Database Syst Rev. 2011b;(3):CD006768. 10.1002/14651858.CD006768.pub2. 10.1002/14651858.CD006768.pub2 PMC4278667 21412896

[93] Schmidt M (1996). Rey auditory verbal learning test: a handbook.

[94] Schneider KJ, Meeuwisse WH, Barlow KM, Emery CA (2018). Cervicovestibular rehabilitation following sport-related concussion. Br J Sports Med.

[95] Schneider KJ, Meeuwisse WH, Palacios-Derflingher L, Emery CA (2018). Changes in measures of cervical spine function, vestibulo-ocular reflex, dynamic balance, and divided attention following sport-related concussion in elite youth ice hockey players. J Orthop Sports Phys Ther.

[96] Silverberg ND, Iverson GL (2011). Etiology of the post-concussion syndrome: physiogenesis and psychogenesis revisited. NeuroRehabilitation.

[97] Silverberg ND, Iverson GL (2013). Is rest after concussion “the best medicine?”: recommendations for activity resumption following concussion in athletes, civilians, and military service members. J Head Trauma Rehabil.

[98] Silverberg ND, Gardner AJ, Brubacher JR, Panenka WJ, Li JJ, Iverson GL (2015). Systematic review of multivariable prognostic models for mild traumatic brain injury. J Neurotrauma.

[99] Silverberg ND, Hallam BJ, Rose A, Underwood H, Whitfield K, Thornton AE, Whittal ML (2013). Cognitive-behavioral prevention of postconcussion syndrome in at-risk patients: a pilot randomized controlled trial. J Head Trauma Rehabil.

[100] Smith A (1991). Symbol digit modalities test.

[101] Spriggs AD, Lane JD, Gast DL (2014). Visual representation of data.

[102] Tate RL, Perdices M, Rosenkoetter U, Shadish W, Vohra S, Barlow DH (2016). The single-case reporting guideline in behavioural interventions (SCRIBE) 2016 statement. Phys Ther.

[103] Tate RL, Perdices M, Rosenkoetter U, Wakim D, Godbee K, Togher L, McDonald S (2013). Revision of a method quality rating scale for single-case experimental designs and n-of-1 trials: the 15-item risk of bias in N-of-1 trials (RoBiNT) scale. Neuropsychol Rehabil.

[104] Tate R, Perdices M (2019). Single-case experimental designs for clinical research and neurorehabilitation settings: planning, conduct, analysis and reporting.

[105] Tator CH, Davis HS, Dufort PA, Tartaglia MC, Davis KD, Ebraheem A, Hiploylee C (2016). Postconcussion syndrome: demographics and predictors in 221 patients. J Neurosurg.

[106] Teo SH, Fong KN, Chen Z, Chung RC (2020). Cognitive and psychological interventions for the reduction of post-concussion symptoms in patients with mild traumatic brain injury: a systematic review. Brain Inj.

[107] Theadom A, Parag V, Dowell T, McPherson K, Starkey N, Barker-Collo S (2016). Persistent problems 1 year after mild traumatic brain injury: a longitudinal population study in New Zealand. Br J Gen Pract.

[108] Thomas E, Fitzgerald M, Cowen G (2020). Does Australia have a concussion ‘epidemic’?. Concussion.

[109] Tomfohr-Madsen L, Madsen JW, Bonneville D, Virani S, Plourde V, Barlow KM (2019). A pilot randomized controlled trial of cognitive-behavioral therapy for insomnia in adolescents with persistent postconcussion symptoms. J Head Trauma Rehabil.

[110] Treleaven J, Peterson G, Ludvigsson ML, Kammerlind A-S, Peolsson A (2016). Balance, dizziness and proprioception in patients with chronic whiplash associated disorders complaining of dizziness: a prospective randomized study comparing three exercise programs. Man Ther.

[111] van der Naalt J, Timmerman ME, de Koning ME, van der Horn HJ, Scheenen ME, Jacobs B (2017). Early predictors of outcome after mild traumatic brain injury (UPFRONT): an observational cohort study. Lancet Neurol.

[112] Vanderploeg RD, Belanger HG, Kaufmann PM (2014). Nocebo effects and mild traumatic brain injury: legal implications. Psychol Inj Law.

[113] Vannest KJ, Ninci J (2015). Evaluating intervention effects in single-case research designs. J Couns Dev.

[114] Wäljas M, Iverson GL, Lange RT, Hakulinen U, Dastidar P, Huhtala H (2015). A prospective biopsychosocial study of the persistent post-concussion symptoms following mild traumatic brain injury. J Neurotrauma.

[115] Ware JE, Sherbourne CD (1992). The MOS 36-item short-form health survey (SF-36): I. Conceptual framework and item selection. Med Care.

[116] Wechsler D (2008). Wechsler Adult Intelligence Scale--Fourth Edition (WAIS-IV).

[117] Wechsler D (2009). Test of premorbid functioning.

[118] Young JE, Beck AT (1980). Cognitive therapy scale.

[119] Zasler N, Haider MN, Grzibowski NR, Leddy JJ (2019). Physician medical assessment in a multidisciplinary concussion clinic. J Head Trauma Rehabil.

[120] Ziino C, Ponsford J (2005). Measurement and prediction of subjective fatigue following traumatic brain injury. J Int Neuropsychol Soc.

