# Supplementary material for: Interdisciplinary Rehabilitation for Concussion Recovery (i-RECOveR): protocol of an investigator-blinded, randomised, case series with multiple baseline design to evaluate the feasibility and preliminary efficacy of a 12-week treatment for persistent post-concussion symptoms
Source: Pilot Feasibility Stud. 2022 Sep 5;8:198. doi: 10.1186/s40814-022-01153-6 (PMC9441831; doi:10.1186/s40814-022-01153-6)
Supplement: Supplementary file 1 — Additional file 1. Informed consent materials. [file 40814_2022_1153_MOESM1_ESM.docx]

Additional file 1: Informed consent materials

**EXPLANATORY STATEMENT**

**Project: Interdisciplinary Intervention for Concussion Recovery (iRECOveR) trial**

**MUHREC Project ID: 23005**

**Investigators: Dr. Catherine Willmott, Dr. Adam McKay**

**Dr. Michael Makdissi, Katie Davies, Prof. Sean Drummond, Professor Jennie Ponsford, Jack Nguyen (PhD Student)**

**Why did you receive this information sheet?**

The Turner Institute for Brain and Mental Health and Neurological Rehabilitation Group are inviting you to take part in a study investigating an interdisciplinary treatment program for persistent post-concussion symptoms. Please read this Explanatory Statement in full before deciding whether to participate in this research. If you would like further information regarding this project, please ask questions of the researcher who provided you with this form.

**Why are we conducting this research?**

Concussion is the most common form of brain injury, making up 75-90% of all brain injuries. Concussions generally resolve within 7-10 days, however, up to 25% of people experience delayed recovery which may interfere with return to pre-injury activities (e.g. work) and cause significant stress for months after injury. Persisting concussion symptoms are thought to reflect a range of factors beyond the original injury to the brain including psychological factors, such as stress and anxiety, and physical factors such as injury to the neck, visual and/or balance systems.

Given that multiple factors may be contributing to the persistence of symptoms after concussion, we aim to evaluate an interdisciplinary intervention that incorporates expertise from psychology, physiotherapy, and medicine.

**What does this research involve?**

**The Interdisciplinary Treatment**

You will receive initial consultations with a psychologist, physiotherapist, and medical doctor. Clinicians will then develop an individualised treatment plan for you, including up to 8 sessions of each of the following specialties. Depending on your needs, you may receive some/all of the treatments below.

Psychology

Changes in mood, emotions and behaviour are common after concussion and may benefit from psychological therapy. Your psychologist will provide education and a type of therapy called cognitive behavioural therapy or CBT. This type of therapy aims to help people develop different ways of thinking and behaving to reduce psychological distress, and has been shown to be helpful for individuals with persistent post-concussion symptoms.

Physiotherapy

Concussion can also result in physical difficulties involving the visual or balance systems, or cervical spine for example. Your physiotherapist will assess your symptoms and provide exercises to treat such problems.

Medicine

You may be offered medical treatment or medication to treat common symptoms such as headache and dizziness.

**Recording**

The psychological sessions will be audio recorded to enable evaluation of this new intervention. These recordings will be password-protected and stored in a secure drive.

**Questionnaires**

You will complete a range of questionnaires before, during, and after treatment in order to track your progress. These questionnaires will assess symptoms of concussion, fatigue, insomnia, health-related quality of life, mood, perceptions about concussion, and your goals, and will take approximately 30 minutes. You will also complete some physiotherapy assessments during these timepoints. The timeline below provides more details. You will also be asked to complete a brief measure of concussion symptoms (2 minutes) online three times a week during the pre-intervention and intervention stage.

One month after the treatment has finished, you will also complete an interview with the researcher in addition to the 30 minutes of questionnaires. You will receive a $50 voucher for completing the interview to reimburse you for your time. Three months after treatment, you will be asked to complete the short 2-minute concussion symptoms questionnaire online.

**Timeline**

| *2-6 weeks* | | | *8-12 weeks* | |  |  |
| --- | --- | --- | --- | --- | --- | --- |
| **Baseline** | **Baseline phase** | **Pre-intervention** | **Intervention phase** | **Post-intervention** | **1-month Follow-up** | **3-month Follow-up** |
| Questionnaires (30 mins), and physiotherapy measures.  Randomisation into baseline phase of 2, 4, or 6 weeks | Online questionnaire (2 mins) three times a week | Questionnaires (30 mins), and physiotherapy measures. | 8-12-weeks of treatment  Online questionnaire (2 mins) three times a week | Questionnaires (30 mins), and physiotherapy measures. | Questionnaires (30 mins), and physiotherapy measures.  1-hour interview | Online questionnaire (2 mins) |

**Why were you chosen for this research?**

You were chosen to participate in this research as you have a history of concussion and have reported post-concussion symptoms which have persisted for more than four weeks

**Source of funding**

Turner Institute for Brain and Mental Health, Monash University.

**Consenting to participate in the project and withdrawing from the research**

Participation in any research project is entirely voluntary. If you/your child do not wish to take part, you/they do not have to. If you/your child decide to take part and later change your mind, you/they are free to withdraw from the project at a later date. Should you/your child withdraw or decline to participate, you/your child will receive treatment as usual with the Turner Clinic, Olympic Park Sports Medicine Centre, and/or Neurological Rehabilitation Group. Alternatively, if we think that participation in the study is having any unforeseen negative consequences for you/your child, you/they will be withdrawn from the study at that time.

**Possible benefits and risks to participants**

Cognitive behavioural therapy, physiotherapy, and medical consultations have been demonstrated to be safe and tolerable for individuals with persistent post-concussion symptoms. Although there are no known risks of participating in these interventions, it is possible that participants may find some of the content during the intervention difficult and/or emotional. The physiotherapy interventions may induce symptoms (e.g. dizziness), however, this will be done in a controlled environment under the care of trained physiotherapists. Research and clinical staff involved in the project are experienced in assessing and managing potential adverse events associated with this treatment and associated outcome measures (e.g., increased anxiety, increased dizziness) should they arise.

There are also several potential benefits of participating in the current study. Participants will be provided with evidence-based treatments and given the skills to cope with and manage their persistent post-concussion symptoms. As a result, they may experience an improvement in their overall mental and physical health. The program is being offered free of charge.

This research will evaluate components of treatment currently being provided by the investigators, and it is hoped that this Monash-led project will eventually establish an evidence-based treatment for concussion that can be rolled out to other concussion services.

**Confidentiality**

Any information obtained in connection with this project and that can identify you will remain confidential and will only be used for the purpose of this research. It will only be disclosed with your permission, except as required by law. If you agree to participate in this project by signing the consent form, we plan to discuss and publish the results of the study. Given the case study design, results may be identifiable to those who know your case well (e.g. doctors, family members). Identifying information, however, will be removed as much as possible.

**Storage of data**

Only the researchers will have access to the study data. Data will be de-identified and securely archived on the Monash University IT server, and paper forms will be stored in a locked cabinet for a minimum of 7 years. It will then be destroyed by shredding or via a contracted secure disposal company.

**Results**

When the project is complete, a general report of the overall study results which does not identify any one individual will be publicly accessible to all interested participants. If you would like to receive a summary of these findings, please email Mr Jack Nguyen at jack.nguyen@monash.edu.

**Ethical Guidelines**

This project will be carried out according to the National Statement on Ethical Conduct in Human Research (March 2007) produced by the National Health and Medical Research Council of Australia. The ethical aspects of this research project have been approved by the Monash University Human Research Ethics Committee.

**Complaints**

Should you have any concerns or complaints about the conduct of the project, you are welcome to contact the Executive Officer, Monash University Human Research Ethics Committee (MUHREC), Room 111, Chancellery Building D, 26 Sports Walk, Clayton Campus Research Office

Monash University VIC 3800

Tel: +61 3 9905 2052 Email: [muhrec@monash.edu](mailto:muhrec@monash.edu) Fax: +61 3 9905 3831

Thank you,

**Chief Investigator**

**Dr. Adam McKay**
Senior Lecturer, Clinic Lead

Neurorehabilitation Clinic

Monash Psychology Centre,

Turner Institute for Brain and Mental Health

Phone: +61 3 9902 4188Email: [Adam.mckay@monash.edu](mailto:Adam.mckay@monash.edu)

**Chief Investigator**

**Dr. Catherine Willmott**

Senior Lecturer, Academic Lead

Neuropsychology & Concussion Clinics,

Monash Psychology Centre,

Turner Institute for Brain and Mental Health

Phone: 0413 545 495
Email: [Catherine.willmott@monash.edu](mailto:Catherine.willmott@monash.edu)

**CONSENT FORM**

**Project: Interdisciplinary Rehabilitation for Concussion Recovery (iRECOveR) Trial**

**Chief Investigators: Dr. Catherine Willmott & Dr. Adam McKay**

**PART A**

|  | Yes | No |
| --- | --- | --- |
| Are you over 18 years of age? | (if answered YES, please proceed to PART C on the next page) | (if answered NO, please fill out PART B AND PART C below) |

**PART B**

I consent to the following: my child/dependent has been asked to take part in the Monash University research project specified above. I have read and understood the Explanatory Statement and I hereby consent for him/her to participate in this project.

| I consent to the following: | Yes | No |
| --- | --- | --- |
| My child/dependent completing the questionnaires at baseline, pre-intervention, post-intervention, and at follow-up appointments. |  |  |
| My child/dependent completing a concussion symptom inventory online three times a week (Monday, Wednesday, Friday) during the baseline and intervention phases. |  |  |
| My child/dependent completing an interview with a researcher at follow-up. |  |  |
| My child/dependent receiving interdisciplinary treatment for concussion from a neuropsychologist, physiotherapist, and medical doctor. |  |  |
| Audio recording during the psychology sessions and follow-up interview |  |  |

Name of Parent/Guardian

Parent/Guardian Signature Date

**CONSENT FORM**

**Project: Interdisciplinary Rehabilitation for Concussion Recovery (iRECOveR) Trial**

**Chief Investigators: Dr. Catherine Willmott & Dr. Adam McKay**

**PART C**

I have been asked to take part in the Monash University research project specified above. I have read and understood the Explanatory Statement and I hereby consent to participate in this project.

| I consent to the following: | Yes | No |
| --- | --- | --- |
| Completing the questionnaires at baseline, pre-intervention, post-intervention, and at follow-up appointments. |  |  |
| Completing a concussion symptom inventory online three times a week (Monday, Wednesday, Friday) during the baseline and intervention phases. |  |  |
| Completing an interview with a researcher at follow-up. |  |  |
| Receiving interdisciplinary treatment for concussion from a neuropsychologist, physiotherapist, and medical doctor. |  |  |
| Audio recording during the psychology sessions and follow-up interview. |  |  |

Name of Participant

Participant Signature Date
